# The Risk Factors Affecting Survival in Colorectal Cancer in Taiwan

**Published:** 2018-04

**Authors:** Chao-Hsien LEE, Shu-Chen CHENG, Hong-Yi TUNG, Shih-Chang CHANG, Ching-Yun CHING, Shu-Fen WU

**Affiliations:** 1. Dept. of Health Business Administration, Meiho University, Pingtung, Taiwan; 2. Dept. of Cancer Registry Division, Cathay General Hospital, Taipei, Taiwan; 3. Dept. of General Surgery, Yuan’s General Hospital, Kaohsiung, Kaohsiung, Taiwan; 4. Dept. of Colorectal Surgery, Division of Surgery, Cathay General Hospital, Taipei, Taiwan; 5. Dept. of Nursing, Yuan’s General Hospital, Kaohsiung, Taiwan; 6. Dept. of Nursing, College of Medicine, I-Shou University, Kaohsiung, Taiwan

**Keywords:** Risk factors, Survival, Colorectal cancer

## Abstract

**Background:**

Colorectal cancer is one of the most common malignancies in developed countries. The incidence of colorectal cancer (CRC) in Taiwan is rising. We aimed to determine the five-yr survival rate of patients diagnosed with CRC and determine factors affecting survival.

**Methods:**

All patients were identified from the Taiwan Cancer Data Base of the Medical Center Hospital in North Taiwan from 2007 to 2013. Data were collected using medical records and the cancer database. In all, 869 patients with CRC were included. Survival analysis was performed using Kaplan-Meier curves, and differences between the curves were analyzed using the log-rank test. Cox proportional hazards regression models were used to analyze survival by each variable.

**Results:**

The five-yr survival rate and the mean survival time after cancer diagnosis were 68.7% and 71.27±1.27 months. Perineural nerve invasion, distant metastasis, age, pathological differentiation grade, obstruction and regional lymph node metastasis were found to be independent predictors of the survival and prognosis of patients with CRC.

**Conclusion:**

Perineural nerve invasion was an important factor related to the survival of CRC patients. Thus, the earlier detection of CRC might help improve survival.

## Introduction

Colorectal cancer (CRC) is the second and third most commonly diagnosed cancer type in females and males, respectively, representing almost 10% of the global cancer incidence. These estimates correspond to age-standardized global incidence and mortality rates of 17.2 and 8.3 per 100000, respectively (1–3). There have been slightly more incident cases and deaths among men than among women in most parts of the world, except in the Caribbean (4). The reported incidence of CRC is highest in developed countries, including the United States, Canada, Australia, northwestern Europe, Japan, South Korea, and Singapore. However, the incidence and mortality rates for CRC are higher in Japan, South Korea, Singapore, China, Hong Kong, Taiwan, and Thailand (5–7). In Taiwan, more than 15410 new cases of CRC were diagnosed in 2013. The incidence rate of CRC is 44.32 per 100000, and the mortality rate is 14.7 per 100000 in both sexes per year (8).

CRC imposes a considerable social economic burden, which includes direct medical care (e.g., treatment by stage at diagnosis, type of cost and disease phase), nonmedical costs, and productivity loss. Cancer survival is an indicator of the overall effectiveness of health services in the management of patients. The five-yr survival rate of individuals with CRC was 65% in the United States. The five-yr survival rate of stage I and II CRC ranges from 80%–90%, whereas stage III and IV metastatic diseases are associated with five-yr survival rates of 60%–71% and 8%–13%, respectively (9,10). Currently, in Taiwan, the overall five-yr survival rate of CRC is 63.0% (11). The five-yr survival rate was 74.3% for stage I CRC compared with 76.6% for stage II, 56.6% for stage III and only 16.7% for stage IV (12). The lifetime cost usually increases with advanced stages. The average cost of CRC in Spain, Iran, and Malaysia was 20.298€, $10715, and RM 13622 for stage I, 28.251€, $1592, and RM 19752 for stage II, 36.8948€, $1642, and RM 24972 for stage III, and 27.001€, $16723, and RM 27377 for stage IV, respectively (7, 13, 14). In Taiwan, the average cost of treating CRC in $/per year was $8416 for stage II, $14334 for stage III, and $21837 for stage IV, indicating large savings with early diagnosis and treatment (6).

CRC is considered primarily a “lifestyle” disease. Demographic variables, such as age, gender, familial CRC history, diets high in calories and animal fat, alcohol consumption, and obesity, in addition to other factors, such as tumor site, size, grade, histologic type, TNM stage, and carcinoembryonic antigen (CEA) level, have all been found to significantly affect survival in CRC (3, 15–19). In the present study, we used population-based data from the Taiwan Cancer Data Base of the medical center hospital in North Taiwan to compare socio-demographic and clinical pathological characteristics, prognostic factors, and overall survival among 3 groups of CRC patients, i.e., those surviving 12, 36, and 60 months.

This study aimed to explore the survival rate and the potential factors influencing survival among CRC patients in northern Taiwan.

## Materials and Methods

### Study population

We conducted a single-center, retrospective cohort study to estimate the survival outcome of patients diagnosed with colorectal carcinoma at Cathay General Hospital in North Taiwan from 2007 to 2013. Data were extracted from medical records and the cancer database by trained data collectors. The eligibility criteria included the following: diagnosis and treatment of CRC; the International Classification of Disease for Oncology, 3rd Edition (ICD-O-3) topographical codes of C18.0-C20.9 (excluding C18.1) and morphology codes of 8000-8152, 8154-8231, 8243-8245, 8247-8248, 8250-8576, 8940-8950 and 8980-8981. Participants who showed more than one type of cancer, ICD-O-3 morphology codes of 8935-8936, 8153, 8240-8242, 8013, 8246, 8249 and 9590-9720, a T_X_N_X_M_X_ stage of 888 or 999, metastasis to the brain, or a survival time of fewer than six months were excluded. Demographic data extracted included gender, age at diagnosis, body mass index (BMI), smoking history, betel nut chewing status, drinking habits, and date of last contact or death. The evaluated tumor characteristics included primary site, histo-logic type, grade/differentiation, and size, as well as treatment type and regional lymph node or distant organ metastases. The disease staging was based on the American Joint Committee on Cancer (AJCC) criteria; cancer site-specific factors included CEA, circumferential resection margin (CRM), tumor regression grade, perineural nerve invasion, KRAS mutation, obstruction, and perforation. Survival data were obtained using death and date of last contact records to determine the current situation or date of death of each patient. The study was reviewed and approved by the hospital’s institutional Review Board (No. CGHP104060).

Statistical analyses were performed using SPSS software (ver. 22.0, Chicago, IL, USA). Quantitative values were compared using t-tests for independent groups. Categorical data were analyzed using the χ^2^ test or Fisher’s exact test. Survival probabilities were estimated at intervals of 12, 36 and 60 months from the date of diagnosis to the date of death. Survival curves were constructed using the Kaplan-Meier method, and differences were analyzed by the log-rank test. Cox proportional hazards regression models were used to analyze survival by each variable. The level of statistical significance was set at *P<*0.05. All reported *P*-values are two-tailed.

## Results

### Sample characteristics

A summary of the demographic and clinical characteristics of the participants is presented in Table 1.

**Table 1:** Demographic and clinical characteristics of CRC patients (N = 869)

***Variable***	***Category***	***N (%)***
Gender		
	Male	454(52.4)
	Female	415(47.76)
Age(yr)		
	Median(range, y)	64(17–97)
	Mean ± SD, y	63.7±0.45
	< 65 yr old	435(50.06)
	≧ 65 yr old	434(49.94)
Primary tumor site		
	Colon	554(63.75)
	Rectum	315(36.25)
Tumor status		
	T1/T2	231(26.58)
	T3	468(53.86)
	T4	170(19.56)
Regional lymph node metastasis		
	N0	476(54.78)
	N1	208(23.94)
	N2	185(21.29)
Regional lymph node involvement		
	No	476(54.78)
	Yes	393(45.22)
Distant metastasis		
	No	747(85.96)
	Yes	122(14.04)
Stage		
	I	190(21.86)
	II	238(27.39)
	III	303(34.87)
	IV	138(15.88)
Histology type		
	Adenocarcinoma	797(91.71)
	Mucinous carcinoma	64(7.36)
	Signet ring-cell carcinoma	8(0.92)
No. of lymph nodes examined		
	< 12	222(25.55)
	≧ 12	647(74.45)
Tumor size		
	< 50 mm	528(65.27)
	≧ 50 mm	281(34.73)
CEA		
	< 5.0 ng/ml	34(3.91)
	≧ 5.0 ng/ml	835(96.09)
CRM		
	Negative	826(94.55)
	Positive	47(5.45)
Perineural invasion		
	No	496(54.68)
	Yes	373(45.32)
KRAS mutation		
	No	43(4.95)
	Yes	25(2.88)
	Unknown	801(92.17)
Obstruction		
	No	512(58.92)
	Yes	357(41.08)
Perforation		
	No	853(98.16)
	Yes	16(1.84)
BMI		
	18.5–24	374(43.04)
	≥24	386(44.42)
	Unknown	109(12.54)
Smoking		
	No	602(69.28)
	Yes	160(18.41)
	Unknown	107(12.31)
Drinking		
	No	642(73.88)
	Yes	122(14.04)
	Unknown	105(12.08)
Chewing betel nut		
	No	733(84.35)
	Yes	30(3.45)
	Unknown	106(12.20)

The follow-up period continued to Dec 2015. We retrospectively evaluated 869 CRC patients from 2007 to 2013. Of these, 454 subjects were males (52.24%). Most patients ranged in age from 51 to 75 yr old (62.37%). The mean and median ages at diagnosis were 63.70 yr and 64 yr, respectively.

Approximately 63.75% of the patients were diagnosed with cancer of the colon. One-third of patients were registered as living with stage III (34.87%) cancer, and the most common histo-pathological type reported was adenocarcinoma (91.71%).

### Survival outcome

**Fig. 1:**
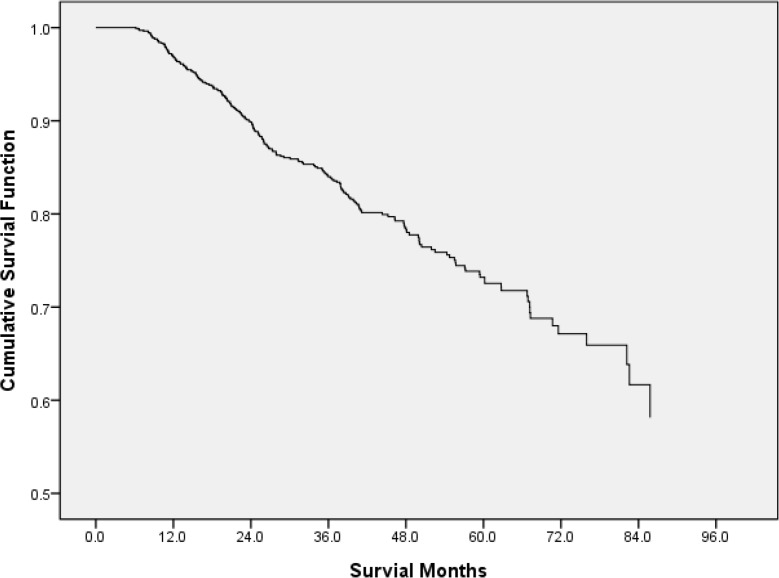
Kaplan-Meier curves of patients with CRC

**Fig. 2:**
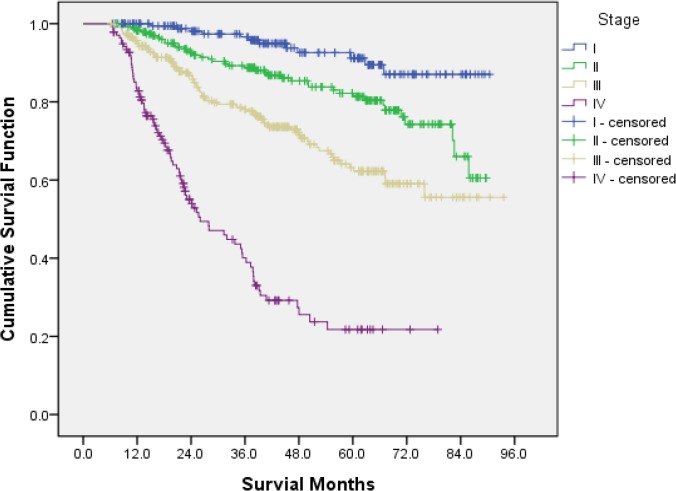
Kaplan-Meier curves of CRC by disease stage

The mean survival time was 71.27±1.27 months. CRC-specific survival was 95.3%, 79.4% and 68.7% at 1, 3 and 5 yr (Fig. 1). The five-yr survival rate for patients with stage I, II, III and IV disease was 91.20%, 82.20%, 63.20% and 21.70%, respectively (Fig. 2).

Log-rank (Mantel-Cox) tests for the equality of the survival functions were conducted as well as a univariate Cox regression analysis. Combining the results of analysis of Log-rank tests and the univariate Cox regression, significant predictors of survival were the age at cancer diagnosis, tumor status, regional lymph node metastasis, distal organ metastasis, cancer stage, pathological differentiation, histopathologic type, tumor size, CRM, perineural nerve invasion, KRAS mutation, obstruction, and perforation (Table 2 and Table 3). The Cox forward stepwise regression model revealed a significant potentially curable disease and risk of CRC death (Table 4).

**Table 2:** Clinical and Pathological variables analysis (N = 869)

***Variable***	***Survival Rate***			***Overall Survival Rate / Std, Mo***	**P-*****value***

	**12 Months**	**36 Months**	**60 Months**		
**Total**	95.30	79.40	68.70	71.27/1.27	
**Gender**					
Male	95.00	80.00	69.90	70.28/1.86	0.981
Female	95.60	78.70	67.60	69.65/1.71	
**Age**					
< 65 yr old	96.90	85.30	76.50	76.74/1.64	< 0.001
≧ 65 yr old	93.70	73.50	60.90	63.78/1.75	
**Primary tumor site**					
Colon	95.50	80.50	69.30	71.90/1.58	0.493
Rectum	94.80	77.60	67.90	67.88/1.95	
**Tumor status**					
T1/T2	99.50	95.60	88.80	83.71/1.49	< 0.001
T3	96.30	80.00	69.20	62.29/1.60	
T4	86.80	55.80	40.70	52.58/3.10	
**Regional lymph node metastasis**					
N0	97.20	89.00	82.50	77.83/1.34	< 0.001
N1	97.60	79.20	63.60	68.97/2.44	
N2	87.90	55.70	39.90	49.71/3.02	
**Regional lymph node involvement**					
No	97.20	89.00	82.50	77.83/1.34	< 0.001
Yes	93.00	68.10	52.10	61.00/2.03	
**Distant metastasis**					
No	97.40	85.20	75.80	76.11/1.26	< 0.001
Yes	82.20	41.30	21.90	36.63/2.68	
**Stage**					
I	100.00	96.60	91.20	84.82/1.54	< 0.001
II	98.30	88.70	82.20	76.59/1.79	
III	95.60	78.10	63.20	68.91/2.21	
IV	82.80	40.10	21.70	36.20/2.56	
**Histology type**					
Adenocarcinoma	95.60	80.90	70.40	72.26/1.31	0.004
Mucinous carcinoma	92.00	63.00	53.00	58.42/4.53	
Signet ring-cell carcinoma	87.50	60.00	30.00	42.41/8.59	
**Pathological differentiation**					
Low grade	96.10	82.10	71.20	72.94/1.31	< 0.001
High grade	88.30	58.00	49.10	54.99/3.79	
**No. of lymph nodes examined**					
< 12	94.00	76.10	68.60	68.77/2.36	0.708
≧ 12	95.70	80.60	68.80	71.55/1.47	
**Tumor size**					
< 50 mm	96.70	82.90	72.40	74.42/1.55	0.003
≧ 50 mm	93.90	75.10	63.70	64.81/2.15	
**CEA**					
< 5.0 ng/ml	100.00	90.00	78.80	73.87/7.01	0.436
≧ 5.0 ng/ml	96.30	80.10	68.70	69.56/1.32	
**CRM**					
Negative	95.60	80.70	70.20	72.41/1.31	< 0.001
Positive	89.40	57.40	45.30	52.78/4.98	
**Perineural invasion**					
No	98.60	92.70	86.60	80.97/1.23	< 0.001
Yes	93.50	66.70	50.10	58.57/2.09	
**KRAS mutation**					
No	90.20	26.70	0.00	25.60/1.79	0.005
Yes	90.00	76.40	40.70	47.86/5.31	
**Obstruction**					
No	96.20	84.50	75.30	73.85/1.42	< 0.001
Yes	94.30	71.30	58.30	64.08/2.17	
**Perforation**					
No	95.30	79.60	69.50	71.62/1.27	0.028
Yes	93.30	68.90	17.20	44.61/6.10	
**BMI**					
18.5–24	96.10	82.70	72.90	71.70/1.56	0.227
≥24	98.30	86.60	75.80	76.91/1.96	
**Smoking**					
No	96.90	83.80	73.90	73.94/1.31	0.646
Yes	97.40	86.30	76.80	71.07/3.16	
**Drinking**					
No	96.80	84.20	73.50	73.74/1.28	0.785
Yes	98.30	85.50	79.50	69.90/3.83	
**Chewing betel nut**					
No	96.90	83.90	73.70	75.09/1.31	0.229
Yes	100.00	96.00	86.40	67.91/2.86	

**Table 3:** Cox regression univariate analysis

***Variable***		***Wald***	***HR***	***95% CI***	**P-*****value***
Age	< 65 yr old				
	≧ 65 yr old	19.85	1.87	1.42–2.47	< 0.001
Tumor status	T1/T2				
	T3	25.03	3.54	2.16–5.82	< 0.001
	T4	68.61	8.74	5.23–14.60	< 0.001
Regional lymph node metastasis	No				
	Yes	58.54	3.05	2.29–4.05	< 0.001
Distant metastasis	No				
	Yes	133.49	5.57	4.16–7.45	< 0.001
Stage	I				
	II	8.14	2.55	1.34–4.86	0.004
	III	27.19	5.01	2.73–9.18	< 0.001
	IV	88.83	18.96	10.28–34.96	< 0.001
Histology type	Adenocarcinoma				
	Mucinous carcinoma	6.96	1.77	1.16–2.71	0.008
	Signet ring-cell carcinoma	4.15	2.80	1.04–7.55	0.042
Pathological differentiation	Low grade				
	High grade	20.25	2.20	1.56–3.10	< 0.001
Tumor size	< 50 mm				
	≧ 50 mm	8.75	1.53	1.15–2.03	0.003
CRM	Negative				
	Positive	13.29	2.18	1.43–3.31	< 0.001
Perineural invasion	No				
	Yes	83.05	4.43	3.22–6.10	< 0.001
KRAS mutation	No				
	Yes	7.22	3.90	1.45–10.51	0.007
Obstruction	No				
	Yes	21	1.87	1.43–2.44	< 0.001
Perforation	No				
	Yes	4.58	2.28	1.07–4.84	0.032

**Table 4:** Forward stepwise Cox regression analysis

***Variable***	***HR***	***95% CI***	***Wald***	**P-*****value***
**Age**				
< 65 yr old				
≧ 65 yr old	2.36	1.76–3.17	32.68	< 0.001
**Pathological differentiation**				
Low grade				
High grade	1.84	1.27–2.66	10.54	0.001
**Perineural invasion**				
No				
Yes	2.90	2.03–4.14	34.26	< 0.001
**Distant metastasis**				
No				
Yes	2.78	2.00–3.87	36.48	< 0.001
**Obstruction**				
No				
Yes	1.38	1.04–1.84	4.94	0.026
**Regional lymph node metastasis**				
No				
Yes	1.81	1.28–2.57	11.22	0.001

The following factors were associated with a relative excess hazard for death: age ≥65 yr (HR = 2.36, 95% CI: 1.76–3.17, *P*<0.001); high grade of pathological differentiation (HR=1.84, 95% CI: 1.27–2.66, *P*=0.001); perineural nerve invasion (HR=2.90, 95% CI: 2.03–4.14, *P*<0.001); metastasis to distant organs (HR=2.78, 95% CI: 2.00–3.87, *P*<0.001); intestinal obstruction (HR=1.38, 95% CI: 1.04–1.84, *P*=0.026); and multiple regional lymph node metastases (HR=1.81, 95% CI: 0.28–2.57, *P*=0.001).

## Discussion

This study observed factors connected with disease survival in a population-wide cohort with access to universal healthcare with a specific focus on recognizing the five-yr survival rate and risk factors of CRC. In a Taiwanese population-based sample of patients with stage I–IV CRC, overall cancer survival reached 71.27±1.27 months. Certain characteristics related to disease progression were strongly associated with the 5-yr risk of death from CRC: age ≥ 65 yr, high grade of pathological differentiation, perineural nerve invasion, distant metastasis, obstruction and multiple regional lymph node metastases each independently increased the risk of death by factors of 1.38 to almost 3. No correlations were found in this study between characteristic variables (e.g., BMI or smoking, drinking, betel nut chewing habits) and CRC survival.

CRC was more common in men than women in our study, which was in agreement with the age-specific incidence rates reported by the Taiwan Health Promotion Administration in 2013. The median age in our study was 64 yr old; however, this age is slightly lower than that observed from cases during 2013, where the mean age of CRC patients was 66 yr (8). The five-yr survival rate found in this study was higher than (12) who reported a survival rate of 55.70% among patients with CRC. This result is probably due to differences in the study populations, as the majority of patients in their study were ≥65 yr old. By age group, the five-yr survival rate was 76.50% in patients younger than 65 and 60.90% in patients ≥ 65 yr old (*P*<0.001). After adjustment with the relevant control variables, we found that being ≥ 65 yr old was associated with a relative excess hazard for death of 2.36 (95% CI: 1.76–3.17, *P*<0.001). Similarly, patient age at diagnosis appears to be an important prognostic factor for all patients (12, 19, 20). In our study, we found that nearly 17% of the patients were younger than 50 yr old, with a minimum age of 17 yr. It is important to note the occurrence of CRC at a young age in our population. Patients younger than 50 yr would not yet qualify for screening and would be more likely to present with symptomatic disease and have a poorer prognosis (21). Therefore, reducing the age at which patients should be screened for this condition could lead to improved outcomes. Such strategies as fecal occult blood testing using immunochemical methods could easily be implemented.

The overall five-yr survival rate in our study was 68.70%; this result is better than that reported by the HPA (8), in Taiwan (12), and the American Cancer Society, which estimated survival rates of 63.0%, 55.69%, and 66%, respectively. In our study, the overall stage-specific five-yr survival rate was 91.20% for stage I, 82.20% for stage II, 63.20% for stage III, and 21.70% for stage IV. Stage I and stage II CRC had an 80%–90% five-yr survival rate, whereas stage III and stage IV metastatic diseases were associated with five-yr survival rates of 60% and 8%, respectively (10). In comparison, the survival rates found in the present study were higher than those previously reported. The risk of death in stage I, II, III, and IV CRC was 2.55 (95% CI: 1.34–4.86, *P*<0.001), 5.01 (95% CI: 2.73–9.18, *P*<0.001), and 18.96 (95% CI: 10.28–34.96, *P*<0.001), respectively. Similarly, the number of lymph node metastases in CRC was an important factor of CRC survival (22, 23). In addition to AJCC stage, other factors influence the survival rate. Thus, the survival rate was investigated pathology results, such as tumor site, size, grade, histology, lymph node metastasis, perineural nerve invasion and other variables in addition to the AJCC stage, T stage, N stage, and M stage as independent variables (20, 22, 24, 25).

In most cases, early-stage CRC does not present obvious symptoms; as such, muscle infiltration or distant metastases have occurred by the time of diagnosis. In this study, tumor status, regional lymph node metastasis, and distant metastasis independently affect the survival rate of CRC patients. These results are consistent with CRC survival rate estimates reported (26–28). The present findings show that high-grade pathological differentiation was associated with a relative excess hazard for death of 1.84 (95% CI: 1.27–2.66, *P*=0.001). Similarly, grade level could independently affect the survival rate of CRC patients (26, 28, 29). Early CRC staging has a positive effect on survival rate. Therefore, the earlier detection of CRC should lead to substantial improvements in survival. CRC screenings were at < 50 yr of age (12, 19, 24).

In the present study, the univariate analysis revealed that histology type was a significant factor. There were significant percentages of mucinous adenocarcinoma (7.36%) and signet ring cell carcinoma (0.92%). These findings are similar to those of previous CRC studies (12, 24, 28). The risk of death in mucinous adenocarcinoma and signet ring-cell CRC with adenocarcinoma was 1.77 (95% CI: 1.16–2.71, *P*=0.008) and 2.80 (95% CI: 1.04–7.55, *P*=0.042), respectively. The histology type of CRC was a risk factor for survival rate (12, 28). However, in the forward stepwise Cox regression analysis, histology type did not independently affect the survival rate of CRC patients.

In this study, perineural nerve invasion was associated with a relative excess hazard for death of 4.43 (95% CI: 3.22–6.10, *P*<0.001). Furthermore, our forward stepwise Cox regression analysis showed that perineural nerve invasion was associated with improved predictions of CRC prognosis, which was in agreement with previous reports (12, 30–32). After adjustment with the relevant control variables, peripheral nerve invasion remained an independent predictor of patient survival and prognosis. The importance of this factor should be considered by clinicians when assessing the prognosis of patients.

Obstruction was a significant factor affecting the survival of CRC patients. In the present study, the univariate Cox regression analysis demonstrated better survival in patients without obstruction (HR = 1.87, 95% CI: 1.43–2.44, *P*<0.001). After adjustment with the relevant control variables, obstruction was associated with a relative excess hazard for death of 1.38 (95% CI: 1.04–1.84, *P*=0.026). These findings are similar to other studies (33, 34).

One limitation of this study was the small sample size; in addition, the findings were generated using data from a single medical center hospital in North Taiwan. Thus, the results of some survival comparisons were not significant. These limitations should be considered when applying these results to other districts in Taiwan that may have demographic differences. Furthermore, multicenter studies should be conducted to merge patient datasets for further research in Taiwan.

## Conclusion

There are numerous prognostic parameters affecting survival in colorectal cancers. Presence of perineural nerve invasion, distant metastasis, age, pathological differentiation grade, obstruction and regional lymph node metastasis are independent predictors of the survival and prognosis of patients with CRC. Perineural nerve invasion and distant metastasis appeared to be important prognostic factors affecting the entire patient cohort. Therefore, the earlier detection of CRC should lead to substantial improvements in survival.

## Ethical considerations

Ethical issues (Including plagiarism, informed consent, misconduct, data fabrication and/or falsification, double publication and/or submission, redundancy, etc.) have been completely observed by the authors.
